# Examining gender: women, health, and the medical profession in Colonial Korea

**DOI:** 10.1590/S0104-59702025000100014

**Published:** 2025-04-07

**Authors:** Wenxin Xu, Michael Shiyung Liu

**Affiliations:** i Lecturer, School of Marxism, Shantou University. Shantou – China, orcid.org/0009-0000-0236-4367, wxxu0215@163.com; ii Distinguished professor, Shanghai Jiao Tong University. Shanghai – China, orcid.org/0000-0002-8617-4495, syliutw@gmail.com

**Figure:**
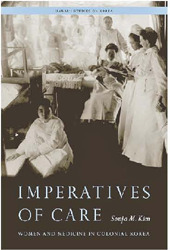
KIM, Sonja M. *Imperatives of care: women and medicine in Colonial Korea*. Honolulu: University of Hawai’i Press, 2020. 240p.

In *Imperatives of care: women and medicine in Colonial Korea*, Sonja M. Kim adopts the lens of gender and adeptly employs diverse archival materials to unfold the narrative of Korean women and modern medicine. Dr. Kim not only brings women who had long been “cast in the shadows of history” (Duby, Perrot, 2023, p.1) to the forefront, but also connects the thoughts and experiences of Korea’s modern medical transformation with broader global processes, in a good response to a previous initiative by this author (Kim, 2010). This groundbreaking English-language text covers topics related to women’s reproductive health, nursing work, family and national transformation in South Korea from the late nineteenth to the early twentieth century.

The book immediately captivates readers with a striking black and white photograph taken in 1907 which shows women in Korean *hanbok,* vividly portraying the diverse and multifaceted roles women assumed in the modernization of healthcare as mothers, patients, nurses, doctors, and midwives. Surprisingly, this riveting cover has gone unnoticed by previous reviewers. It signified the shift for women from a social role to a social gender, while seamlessly introducing the central theme of the book: Korean women actively asserting their agency in the late nineteenth and early twentieth centuries against the intricate background of “Korean nationalism, Japanese imperialism, and Christian mission evangelism” (Kim, 2020, p.10), playing a pivotal role in advancing the modernization of Korean medicine and healthcare sciences. Kim’s research delves into how women navigated the constrained spaces of medicine and draws attention to this group, which seldom voiced its experiences.

The book is structured into four chapters that address issues such as women’s education and health, women’s medical professions (doctor, nurse, and midwife), and gender politics in Korea from the Korean Empire (1897-1910) to the Japanese colonial period (1910-1945), revealing another facet of Korean healthcare modernization. In reading this book, one might anticipate a positive resolution despite the pressures of war, revolution, colonization, power dynamics, and gender oppression; however, in the concluding section the author prompts readers to recognize that it is not a simplistic tale of “oppression and resistance” but rather one that left behind “a mixed legacy” (Kim, 2020, p.138). Even in contemporary times, a prominent feature of healthcare services in Korea has been the continual prioritization of women’s “reproductive and child-rearing capacities” (p.137). Kim lays bare the ongoing narrative, encompassing issues of women’s status, autonomy, careers, families, and societal interactions. The author refrains from presenting conclusive answers, and instead encourages readers to focus on marginalized groups and to confront and contemplate the persistent gender issues in today’s society.

There were several distinctive features in this book. First, it employed a diverse range of primary source materials including newspapers and journals, diaries (such as Horace Allen’s diary), textbooks (for example, of elementary ethics), official government reports, and memoirs (like *Kil Chǒnghǔi* and *Na ǔi chasǒjǒn*), the first time some of these sources were introduced in historical research. The author also adeptly utilized translation and conceptual analysis. For example, in the case of *ǔsaeng* (traditional Korean doctors), Kim illustrates the transition from traditional to modern medicine as a nuanced process, depicting traditional medicine as a dynamic process of change and form of cultural and social construction. Furthermore, despite starting from a gender perspective, the author takes a holistic view of society, asserting that “women’s history is inevitably also men’s history” (Duby, Perrot, 2023, p.1). The text also addresses issues related to the transformation of Korean society, evolution of medical knowledge, and themes of nationalism and imperialism from the late nineteenth and early twentieth centuries, contributing to the study of Korean medical history and enriching discussions in Korean and broader Asian studies.

Despite these positive attributes, certain aspects could be enhanced. The author adeptly delves into the interaction between women and the contemporary political and social milieu, but an exploration of women’s introspection regarding their bodies, sexuality, sexual orientation, and emotional dimensions is notably absent. Introducing facets of female emotions such as varied feelings towards their bodies could add depth and vibrancy to the characters portrayed. Additionally, questions arise within the dynamics of ideological clashes (between Japanese colonialism and missionary evangelism, for example) about how women navigated interactions amongst themselves and with men. In other words, questions of how female healthcare professionals of different races or the same race relate to each other, and how they perceive each other amid these ideological struggles, also require clarification.

While the author meticulously explored the evolution of concepts, one indispensable aspect that the book should address is how the experiential dimension of “practice” influences the acceptance and adaptation of these concepts. For instance, after 1910, Japanese colonial authorities in Korea gradually implemented an education model centered on “clinical practice” (Kim, 2020, p.60), which deviated from the cultivation of academically-oriented doctors. Investigating specific practices in clinical settings, such as changes in equipment, attire, technical tools (e.g., midwives’ delivery kits), surgical instruments, and operational guidelines is consequently crucial: how did these changes in tools impact medical ideologies or gender constructions? Considering the wealth of materials in the study of colonial medical history, the author could offer a more nuanced and insightful exploration in this domain.

Despite the existence of several insightful studies, the current body of research examining the themes of colonial medicine in Latin America remains insignificant and conspicuously lacks the incorporation of gender perspectives. As such, Iona McCleery’s definition of colonial medicine as “practiced in colonies, connected to the colonizing powers” endures (Ramos, 2021), while discourses predominantly favor masculinist viewpoints. The dearth of scholarly examination through a gendered lens, as Kim does here, constitutes a consequential lacuna that necessitates further probing to foster a more comprehensive grasp of the medical praxes and socio-cultural dynamics that prevailed during the colonial era in the region. In sum, this pioneering work has already established a vital foundation and Kim’s work can serve as an impetus for both Korean and Latin American studies of colonial medicine.
